# Efficiency of Fine-Needle Aspiration (FNA) in Relation to Tru-Cut Biopsy of Lateral Neck Swellings

**DOI:** 10.7759/cureus.64224

**Published:** 2024-07-10

**Authors:** Mohammed S Al Olaimat, Fahad S Al Qooz, Zaid R Alzoubi, Elham M Alsharaiah, Ali S Al Murdif, Mohammad O Alanazi

**Affiliations:** 1 Maxillofacial Surgery, King Hussein Medical Center, Amman, JOR; 2 Pathology, King Hussein Medical Center, Amman, JOR; 3 Maxillofacial Surgery, King Abdulaziz Airbase Military Hospital, Dhahran, SAU

**Keywords:** tru-cut biopsy, lateral neck mass, aspiration, fine needle, neck

## Abstract

Background

Lateral neck masses have always been difficult to diagnose without proposing a differential diagnosis. Fine-needle aspiration (FNA) was proposed to be a cost-effective method and less invasive than a tru-cut biopsy and may provide a provisional diagnosis in relation to cytopathology. FNA has also been shown to improve the diagnosis of neck masses such as cervical lymphadenopathy, neck cysts, and parotid masses, whether malignant or benign. This study aims to evaluate the accuracy of FNA cytopathology versus a tru-cut biopsy histopathological examination.

Materials and methods

This study was conducted retrospectively in King Hussein Medical Hospital, Royal Medical Services, Hashemite Kingdom of Jordan, from January 2019 to January 2024. Ethical approval was taken to conduct this study with reference number 06/2024. All patients included in this study have given verbal and written consent to perform FNA and surgical tru-cut biopsy. The inclusion of patients was based on any person above the age of 16 who underwent an FNA followed by a surgical biopsy to correlate with the primary diagnosis. Exclusion criteria involved any patient who missed one of the above criteria. Statistical analysis was performed using IBM SPSS v29 (IBM Corp., Armonk, NY, US) with significant results considered with a p-value <0.05.

Results

A total of 107 patients were included in this study. A correlation between FNA results and final histopathological biopsy was done with an accuracy of 90.6%, specificity of 94.3, predictive positive value of 73.6%, and negative predictive value of 94.3%. There was a statistical significance between FNA and tru-cut biopsy with a p-value of <0.001.

Conclusion

FNA is a great tool to consider when diagnosing lateral neck swellings. Since it was statistically significant, FNA should be considered for any lateral neck swelling before any surgical tru-cut biopsy for a definitive diagnosis.

## Introduction

Horvath et al. investigated the accuracy of ultrasound (US) alone and reported higher accuracy for ultrasound-guided (USG)-FNA of head and neck lesions. When FNA alone was done, it was found to be superior to US alone [1]. A study on the usefulness of FNA in the management of parotid masses reported high predictive positive and negative values in detecting salivary gland pathology [2]. FNA has become a very well-known tool in the diagnosis of lateral neck swellings. It is an easy diagnostic tool, cost-effective, and minimally invasive. Lateral neck swellings include benign or malignant neoplastic changes. The FNA was proposed years ago in relation to preventing any seeding of tumor cells while undergoing a true incisional biopsy. It has been proposed to report a low specificity percentage for malignant transformation [2-6]. In a cytopathologic study to diagnose adenoid cystic carcinoma of the parotid gland, the final biopsy revealed that FNA reported an accuracy of 90% [3]. Akhavan-Moghadam et al. studied FNA results for head and neck masses and reported accuracy, sensitivity, and specificity to be 92.3%, 95%, and 85%, respectively [4]. Alwagdani et al. investigated the role of FNA in detecting malignant tumors and found the accuracy/sensitivity to be 85.7%, and specificity 93.8% with a positive predictive value of 75% [5]. A study conducted by Marzouki et al. investigated 43 salivary gland cases that underwent parotidectomies, of which 38 FNA results were conclusive [7]. The study found that specificity for salivary gland FNA was 100%, and sensitivity was 50% with a positive predictive value of 100% [7]. FNA has been proposed as the initial diagnostic tool for salivary gland tumors, whether benign or malignant. Only a few complications have been reported when performing FNA, they are hemorrhage, nerve damage, and vasovagal complications. Branchial cleft cysts with the aid of FNA have been shown to report epithelial cells with subepithelial lymphoid aggregates [8]. Ultrasound-guided FNA (USG-FNA) has a similar specificity and sensitivity to contrast-enhanced computed tomography (CECT) in detecting metastatic carcinoma [9].

Cengiz et al. evaluated parotid masses that underwent preoperative FNA or core needle biopsy (CNB) versus surgical intervention [10]. The study found that the sensitivity of a parotid gland tumor diagnosis was 40% and specificity was 100%[10]. A study evaluating core-needle biopsy versus tru-cut biopsies in salivary gland malignancies revealed a negative predictive value, positive predictive value, and accuracy of 94%, 90%, and 92%, respectively [11]. Another study to evaluate the accuracy of fine-needle aspiration cytology (FNAC) versus CNB of parotid tumors whether benign or malignant found that out of 126 patients who underwent FNA alone, FNA with CNB, or CNB alone, 62 patients had a history of cancer of which 6 had primary salivary gland malignancies. They illustrated that out of 126 patients, 122 FNAC results were adequate and 13 were categorized as non-diagnostic. Out of these 122 specimens, 41 specimens were included for salivary gland tumors and had a sensitivity of 100% and a specificity of 92.3% in detecting malignant transformation in salivary glands [12]. A study was conducted by Zaifullah et al. to diagnose and treat branchial cleft anomalies [13]. They included 12 cases out of 26 and proposed that only 5 cases were diagnosed on presentation correctly. The other seven cases were misdiagnosed as cervical abscesses, tuberculosis, or inclusion cysts. Their study proposed that a USG-FNA would have aided in overcoming unwanted diagnoses [13]. Pynnonen et al., in their study to evaluate neck masses, stated that clinicians should perform FNA instead of open biopsy with patients who present with a neck mass and are thought to be at substantial risk of malignancy. In their practice guidelines, they found that FNA is the preferred method of diagnosing cancer in a presenting neck mass [14]. Patients with human-papilloma-virus-positive squamous cell carcinoma (HPV-positive SCC) usually present with a cystic appearance on imaging, thus making the FNA a valuable tool to decrease diagnostic delays, complications, and unwanted open biopsies [14]. Although open biopsy is not indicated in salivary gland diseases, FNA has been shown to have a high accuracy rate in detecting lymphadenopathy such as metastatic malignancy, infectious diseases, and granulomatous lymphadenitis. Yet, the role of FNAC in initially diagnosing primary lymphoid malignancies remains unclear [15-18]. Rammeh et al. studied 1262 cases for FNAC accuracy for head and neck masses and reported that only 265 results were included with cytohistological correlation [19]. In their study, lymph node FNAC sensitivity and specificity were 98.2% and 100%, respectively, for the detection of malignancy. The sensitivity and specificity of salivary gland FNAC were 66.7% and 100%, respectively, in diagnosing malignancies of salivary gland origin [19]. This study aims to evaluate the accuracy and sensitivity of FNA versus biopsy of patients presenting with expansive nature neck masses.

## Materials and methods

This study was conducted retrospectively on all patients who underwent FNA involving a persistent non-thyroid lateral neck swelling followed by a surgical intervention, whether incisional or excisional biopsy. The study was carried out in King Hussein Medical Center, Royal Medical Services, Hashemite Kingdom of Jordan, from January 2019 to January 2024.

Whether ultrasound-guided or not, FNA was performed by pathologists. The needle gauge used in the procedure was 25-gauge. The collected samples were placed on glass slides and were fixed and further stained with hematoxylin & eosin (H&E). Final FNA results were correlated with the final tru-cut biopsy diagnosis. Tru-cut biopsies were performed by maxillofacial surgeons, and all specimens were sent to histopathology for further evaluation.

True positive results indicated that FNA showed atypical cells and revealed a true malignancy on the final biopsy. True negatives were any mass that was deemed non-suspicious and reported as such whether the diagnosis was inflammatory, infectious, cystic, or benign. False positives were considered on the basis that the preliminary diagnosis was suspicious and reported the opposite. False negative was considered as previously mentioned but reported a malignant diagnosis. Sensitivity was calculated with True Positive/(True Positive + False Negative), specificity; True Negative/(True Negative + False Positive), positive predictive value; True Positive/(True Positive + False Positive), negative predictive value; True Negative/(True Negative + False Negative), and accuracy; (True Positive + True Negative)/ALL.

All patients included in the study have given verbal and written consent to perform FNA and to undergo surgical procedures in accordance with hospital policy. The study was approved by the Ethical & Research Committee at King Hussein Medical Hospital and was assigned reference number 06/2024. All data were collected from our facility’s histopathological and surgical registry and were analyzed in the Microsoft Excel (Microsoft Corporation, Redmond, WA, US) database. The search engine used was PubMed/Medline, with keywords used ‘fine needle’, ‘aspiration’, and ‘neck’.

The inclusion criteria included any patient above the age of 16 with a history of non-thyroid lateral neck swelling that was deemed adequate in size for an FNA. Any patient who underwent an FNA followed by a surgical procedure was included in this study. The exclusion criteria involved any patient who had a neck swelling but did not undergo an FNA; any patient who underwent an FNA procedure but did not undergo the surgical procedure; as well as all inadequate and suboptimal FNA samples.

Statistical analysis was performed using IBM SPSS v29 (IBM Corp., Armonk, NY, US). Statistical significance was defined as p-value < 0.05. Variables were analyzed via the chi-square.

## Results

A total of 145 cases involved lateral neck swellings, out of which 107 patients were included in the study. FNAC results in relation to surgical intervention showed that the accuracy of the procedure was 90.6%, specificity was 94.3% with a predictive positive value, sensitivity was 73.6%, and negative predictive value was 94.3%. A total of 83 results were considered true negative (TN), 14 were true positive (TP), 5 were false negative (FN), and 5 were false positive (FP).

Eighteen FNAs reported atypical cells that underwent immediate intervention to rule out malignant transformation yet ended with a final decision of being malignant in nature, out of which four biopsies reported negative for malignancy. Sixty-seven FNAs from salivary gland masses reported the most common tumor was pleomorphic adenoma (40/67) followed by warthin tumors (18/67). Five FNAs reported pleomorphic adenoma as a primary diagnosis reported positive for malignancy. Three of those five reported low-grade acinic cell carcinoma, one was polymorphous low-grade adenocarcinoma, and the last was reported as large B-cell lymphoma. Twenty-two FNAs were reported from other sites of the neck, with the most common being a branchial cleft cyst (7/22). Only one patient result reported adenoid cystic carcinoma. The second most common diagnosis was reactive lymph nodes (4/22) followed by lipoma (3/22).

**Figure 1:**
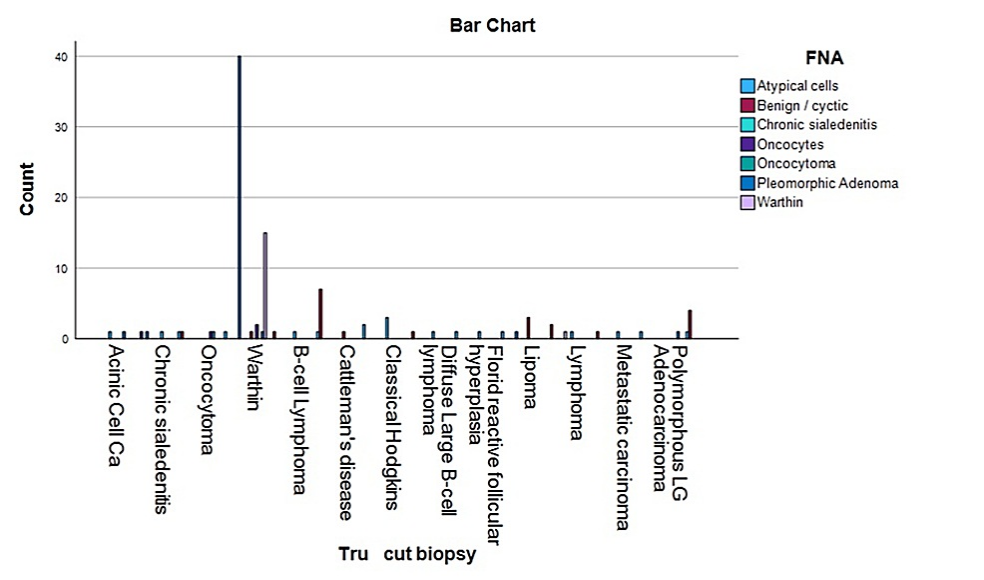
Descriptive analysis correlation between FNA and tru-cut biopsy FNA: fine-needle aspiration

Statistical analysis was done on the total sample size and was statistically significant with a p-value of <0.001 (Figure 1). On FNA, 89 out of 107 were benign and the remainder were suspected of malignancy. On the tru-cut biopsy, 88 out of 107 reported benign. The most common benign mass met of all cases was pleomorphic adenoma (p < 0.001). The most common malignant disease was acinic cell carcinoma (p < 0.001).

Out of 145 cases, 6 patients had inadequate, or suboptimal, FNA results. Out of these patients, three reported salivary gland malignancies, of which two patients had mucoepidermoid carcinoma and one patient had adenoid cystic carcinoma. The three remaining cases were benign. All six patients underwent proper management and care in relation to their incisional biopsies.

## Discussion

The main purpose of FNA for lateral neck swellings is to differentiate whether the mass is benign in nature or suspicious of malignancy. A primary diagnosis decreases the risk of unnecessary surgical procedures before a proper diagnosis is contemplated. In this study, FNA results showed statistically significant results when compared to final histopathologic results. In our study, we have undertaken the following procedure to correlate with evidence present in the literature. Diagnostic accuracy, specificity, and sensitivity were 90.6%, 94.3%, and 73.6%, respectively. Consistent with our results were accuracy, specificity, and sensitivity in the studies of Wan Y, Akhavan-Moghadam J, and Alwagdani A [3-5]. Other studies showed different accuracy, specificity, and sensitivity results. A cytological examination is one of the prerequisites of neck masses. With a high positive predictive value for neck masses, and high sensitivity and accuracy, we propose considering FNA to be a tool to be used in the diagnosis and not as an adjunct [1,10-13]. FNA can differentiate between suspicious masses being benign or malignant and is highly dependent on the cytopathologist's experience [1]. FNA has been performed, either US-guided or without, by well-trained cytopathologists in an outpatient setting. With the help of ultrasound, many masses were visualized accurately.

We have linked FNA and tru-cut biopsy even with a small sample size. It has been shown that FNA can be considered prior to performing any surgical procedure on a lateral neck swelling. A few studies have involved all neck masses together as in our study. Most studies involved FNA accuracy and biopsy of salivary gland tumors. Results from our study thus show that salivary gland tumors are the most commonly encountered, with pleomorphic adenoma being the most common presentation of neck masses as a vast majority. Although neck masses present concomitantly, we focused more on the bigger picture, that is, the neck masses. Our study considers all neck masses to need an FNA regardless of the nature of the presentation.

**Figure 2:**
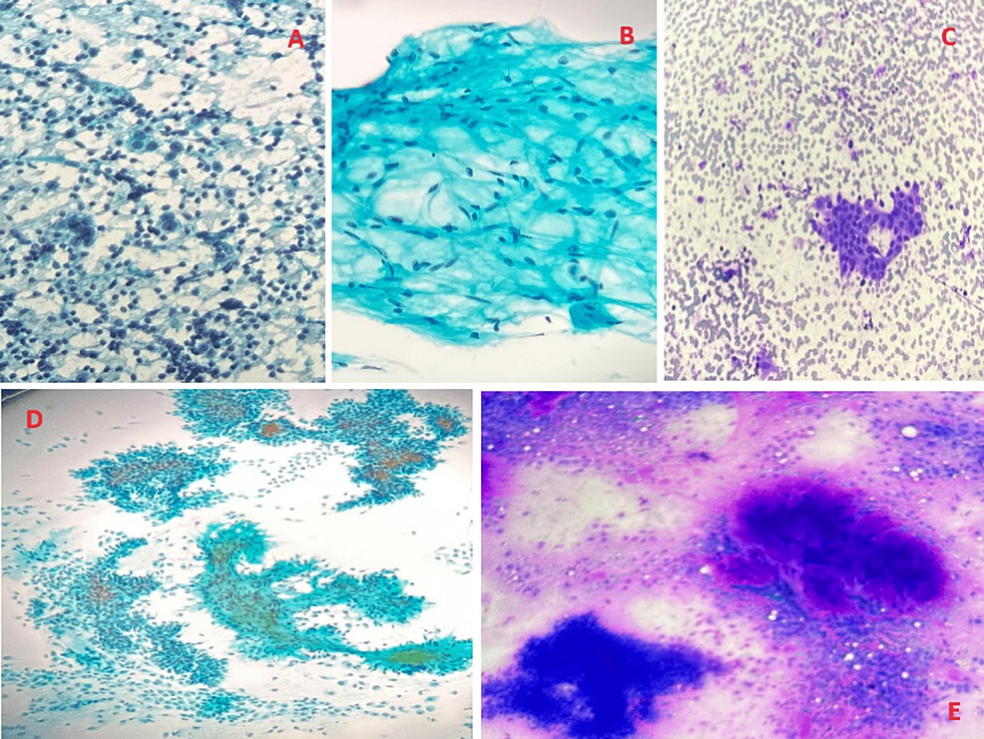
Fine-needle aspiration cytology samples of multiple neck pathologies A. Diffuse large B-cell lymphoma (DLBCL) cytology showing large atypical lymphoid cells with vesicular chromatin and prominent nucleoli. B. Fine-needle aspiration cytology showing adipocytes (x40). C. Fine-needle aspiration cytology showing cohesive groups of oncocytic cells with dense cytoplasm in a background with lymphocytes. D. Papanicolaou stain: pale, stained fibrillary stroma. E. Neoplasm, benign, pleomorphic adenoma, salivary gland FNA: benign myoepithelial cells intermingled with a fibrillary magenta-colored matrix

**Figure 3:**
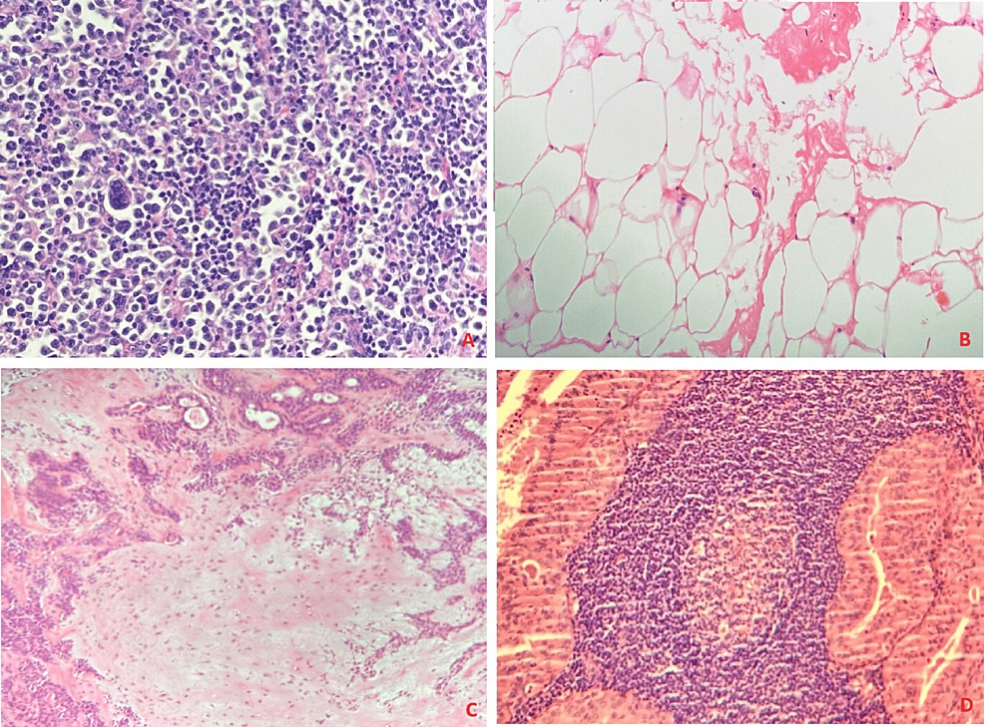
Histologic samples of different pathologic entities A. Diffuse large B-cell lymphoma, NOS. Effacement of the normal tissue architecture by a diffuse infiltrate of large atypical lymphoid cells. B. Lipomas are composed of mature adipose tissue. Adipocyte nuclei are small and round, often compressed at the periphery of the cell. C. Pleomorphic adenoma is a triphasic tumor with ductal (epithelial), myoepithelial, and chondromyxoid stromal components. D. Warthin tumor showing papillary structures lined by bilayered oncocytic epithelial cells and surrounded by a lymphoid stroma.

Also, since FNA is a cheap and non-invasive procedure, we highly recommend that an FNA is performed before any neck mass surgical intervention [5]. FNAC cytology puts forward the advantage of being cost-effective and less invasive when done but the drawback of such a procedure is that the cytological smear cannot differentiate between cells due to the limited amount of cells being examined. It has been suggested in the literature that FNA has a high value and accuracy with high rates of accurate diagnosis. Based on the pathological examination, the pathologist makes a diagnosis based on the data that has been examined (Figures 2, 3).

The tru-cut biopsy, whether incisional or excisional, is performed in an inpatient setting by maxillofacial surgeons. The surgical procedures included incisional or excisional biopsies. Surgical procedures are invasive and place patients at risk of complications such as nerve impairments, surgical site infections, fistula formation, etc. A primary FNA diagnosis allows the surgeon to discuss treatment options with the patient prior to going for further invasive treatment.

Limitations of the study

The study is limited only to a few cases that were diagnosed over a small period of time. The retrospective design of the study is one of the highest limitations after sample size. Many patients were excluded from this study due to the inadequacy of samples related to FNA or if the patients had not undergone surgical procedures. The use of ultrasound guidance for all FNA biopsies is one of the drawbacks that we could not assess. More research needs to include all neck pathologies in bigger research. More correlations are needed to increase the awareness of the use of FNA before intervening surgically for either an incisional or an excisional biopsy.

## Conclusions

FNA has always been an additive tool in diagnosing neck masses. The use of FNA is highly recommended for any neck mass, and it was found to be statistically significant. Its use is easy, cost-effective, and less invasive. Its accuracy reaches above 90% in the diagnosis of masses of the neck, thus making it a particularly valuable tool for a clinician. However, an FNA resulting in atypical cells mandates an urgent intervention to rule out malignancy and metastases.
